# A large deletion in the *GP9* gene in Cocker Spaniel dogs with Bernard-Soulier syndrome

**DOI:** 10.1371/journal.pone.0220625

**Published:** 2019-09-04

**Authors:** Fabio Gentilini, Maria Elena Turba, Fiorella Giancola, Roberto Chiocchetti, Chiara Bernardini, Markéta Dajbychova, Vidhya Jagannathan, Michaela Drögemüller, Cord Drögemüller

**Affiliations:** 1 Department of Veterinary Medical Sciences, University of Bologna, Bologna, Italy; 2 Genefast, Forlì, Italy; 3 Genomia, Plzen, Czech Republic; 4 Institute of Genetics, Vetsuisse Faculty, University of Bern, Bern, Switzerland; University of Kentucky, UNITED STATES

## Abstract

Inherited bleeding disorders including abnormalities of platelet number and function rarely occur in a variety of dog breeds, but are probably underdiagnosed. Genetically characterized canine forms of platelet disorders provide valuable large animal models for understanding similar platelet disorders in people. Breed-specific disease associated genetic variants in only eight different genes are known to cause intrinsic platelet disorders in dogs. However, the causative genetic variant in many dog breeds has until now remained unknown. Four cases of a mild to severe bleeding disorder in Cocker Spaniel dogs are herein presented. The affected dogs showed a platelet adhesion defect characterized by macrothrombocytopenia with variable platelet counts resembling human Bernard-Soulier syndrome (BSS). Furthermore, the lack of functional GPIb-IX-V was demonstrated by immunocytochemistry. Whole genome sequencing of one affected dog and visual inspection of the candidate genes identified a deletion in the *glycoprotein IX platelet* (*GP9*) gene. The *GP9* gene encodes a subunit of a platelet surface membrane glycoprotein complex; this functions as a receptor for von Willebrand factor, which initiates the maintenance of hemostasis after injury. Variants in human *GP9* are associated with Bernard-Soulier syndrome, type C. The deletion spanned 2460 bp, and included a significant part of the single coding exon of the canine *GP9* gene on dog chromosome 20. The variant results in a frameshift and premature stop codon which is predicted to truncate almost two-thirds of the encoded protein. PCR-based genotyping confirmed recessive inheritance. The homozygous variant genotype seen in affected dogs did not occur in 98 control Cocker Spaniels. Thus, it was concluded that the structural variant identified in the *GP9* gene was most likely causative for the BSS-phenotype in the dogs examined. These findings provide the first large animal *GP9* model for this group of inherited platelet disorders and greatly facilitate the diagnosis and identification of affected and/or normal carriers in Cocker Spaniels.

## Introduction

Sporadic cases of a severe bleeding disorder characterized by dysfunctional platelets could be explained by rare forms of inherited thrombocytopathies [1]. This group of haemorrhagic disorders show a marked phenotypic heterogeneity classified according to platelet function into adhesion, activation, secretion, and aggregation defects [2]. The two best characterized platelet adhesion defects in humans are Glanzmann thrombasthenia (GT, OMIM 273800) and Bernard-Soulier syndrome (BSS, OMIM 231200) [3,4]. Human BSS is a rarely reported hereditary bleeding disorder initially described by Bernard and Soulier in 1948 in a young man with a prolonged bleeding time, mild thrombocytopenia and giant platelets approaching the size of lymphocytes caused by a defect of the platelets lineage [5,6]. BSS often presents early with bleeding symptoms, such as epistaxis, ecchymosis, menometrorrhagia, and gingival, gastrointestinal, muscular or visceral bleeding. BSS is caused by a defect in or deficiency of the platelet membrane von Willebrand factor (vWF) receptor complex, glycoprotein Ib-IX-V (GPIb-IX-V) [7]. At sites of vascular injury, the GPIbα component of this receptor binds the adhesive protein, vWF, to support platelet adhesion and platelet thrombus formation. Since GPIb-IX-V is composed of four subunits encoded by four separate genes, glycoprotein Ib platelet alpha subunit (*GP1BA*), glycoprotein Ib platelet beta subunit (*GP1BB*), glycoprotein IX platelet (*GP9*), and glycoprotein V platelet (*GP5*), the molecular basis of BSS is characterized by locus and allelic heterogeneity [8], although, until today no disease causing the *GP5* variant has been identified.

Moreover, various forms of inherited platelet disorders have been reported in dogs at the functional, biochemical, and molecular levels [9]. Currently, the Online Mendelian Inheritance in Animals (OMIA) [10] catalogue reports three genetically characterized canine forms of Von Willebrand disease (OMIA 001057-9615, OMIA 001339-9615; OMIA 001058-9615), corresponding to the most common hereditary extrinsic platelet disorder in humans in which the platelets are normal but a large multimeric glycoprotein necessary for their function is either absent, reduced, or dysfunctional (OMIM 613160). On the other hand, intrinsic platelet disorders also occur sporadically in dogs and, involve the platelets directly arising from abnormalities in platelet granules, membrane glycoproteins, signal transduction proteins, or proteins involved in platelet production from megakaryocytes [9]. Currently, OMIA lists the following causative breed-specific genetic variants of eight different canine intrinsic platelet disorders: *ITGA2B*-associated forms of thrombasthenia in Great Pyrenees and Otterhounds corresponding to human GT (OMIA 001000-9615), the *AP3B1*-associated platelet storage pool defect in Grey Collies (OMIA 000248-9615), *RASGRP1*-associated platelet aggregation defects in Basset hounds, Eskimo Spitz and Landseers (OMIA 001003-9615), *P2RY12*-associated platelet aggregation defect in Greater Swiss Mountain dogs (OMIA 001564-9615), *FERMT3*-associated leukocyte adhesion deficiency in German Shepherds (OMIA 001525-9615), *TUBB1*-associated macrothrombocytopenia in Cavalier King Charles Spaniels and nine other dog breeds (OMIA 001001-9615), *MYH9*-associated giant platelet disorders in Pugs (OMIA 001608-9615), and *ANO6*-associated platelet activation defect in German Shepherds (OMIA 001353-9615). However, the list of canine bleeding defects in which the molecular etiology was not investigated is still long and includes not only predominantly extrinsic platelet disorders (OMIA 001056-9615) but also rare forms of intrinsic platelet disorders such as the inherited platelet delta-storage pool disease described in Cocker Spaniel (CS) dogs (OMIA 001198-9615).

In the present study, three independent Cocker Spaniels of Italian origin and a fourth dog of Czech origin, which all showed characteristic signs of human BSS, were investigated. An earlier report described a similar platelet disorder in five Cocker Spaniels of American origin [11], but the molecular etiology was not investigated in any of these cases. The aim of the present study was to identify the causative genetic variant for the phenotype in the affected Cocker Spaniel of European origin.

## Materials and methods

### Ethics statement

This study did not require official or institutional ethical approval as it was not experimental. The animals were handled according to good ethical standards and Italian legislation. The dogs in this study were examined with the written consent of their owners. The aim was to identify the cause of the congenital disorder and thereby improve the animal welfare situation of the breed.

### Brief clinical description of cases

Proband BSS#1 was represented by a three year-old CS male dog visited by the practitioner due to a prolonged episode of bilateral epistaxis. The dog underwent rhinoscopy with unrewarding findings and was then referred to the Veterinary Hospital. The owner did not report any previous history of hemorrhagic diathesis. At admission, the presenting complete blood count (CBC) values were indicative of a mild thrombocytopenia (56 x 10^3^ /μL; reference range 160–500 /μL) with a majority of giant platelets. The Mean Platelet Volume (MPV) was 27.3 fL (ADVIA 2120, Siemens; reference value 8.7–15.0 fL). The hematocrit (Hct) (37.3%) was within the reference range (37.0–55.0%). The dog was maintained under general anesthesia and underwent supportive therapy (S1 Fig). Despite the therapy, the overall conditions worsened with the onset of hematuria and melena and a dramatic drop in Hct. The dog was transfused with whole blood twices and immunosuppressive therapy was instituted until the clinical signs gradually resolved, within 10 days. Months after the bleeding episode, the dog underwent another CBC as a control. Despite an apparent healthy condition, the CBC continued to show alterations inconsistent with ongoing immune-mediated thrombocytopenia (IMT). After the above-described episode, no other severe bleeding episode occurred in the next 3 years, and the only complaint of the referring practitioners was a mild but continuing delay in clot formation after venipuncture with the tendency of hematoma formation at the venipuncture site.

Proband BSS#2 was a two year-old male CS which was referred for a mild but persistent bleeding episode after neutering. The CBC showed macrothrombocytopenia. The macrotrombocytopenia persisted after resolution of the clinical signs with fluctuating platelet values in the 20K–60K range.

Proband BSS#3 was represented by a three-year-old male CS with periodic and self-limiting episodes of gingival bleeding associated with macrothrombocytopenia unresponsive to corticosteroid drugs.

Proband BSS#4 was a female CS of Czech lineage which had a history of a few cases of bleeding problems. The dog had recurrent mild to moderate bleeding problems including gingival bleeding, hematuria and vulvar bleeding after mating. In addition to BSS#4 in this pedigree, another relative bitch died of severe bleeding episode after delivery and despite transfusion and symptomatic therapy.

### Phenotypic characterization

#### Complete blood count and platelet analysis

**Fig 1:**
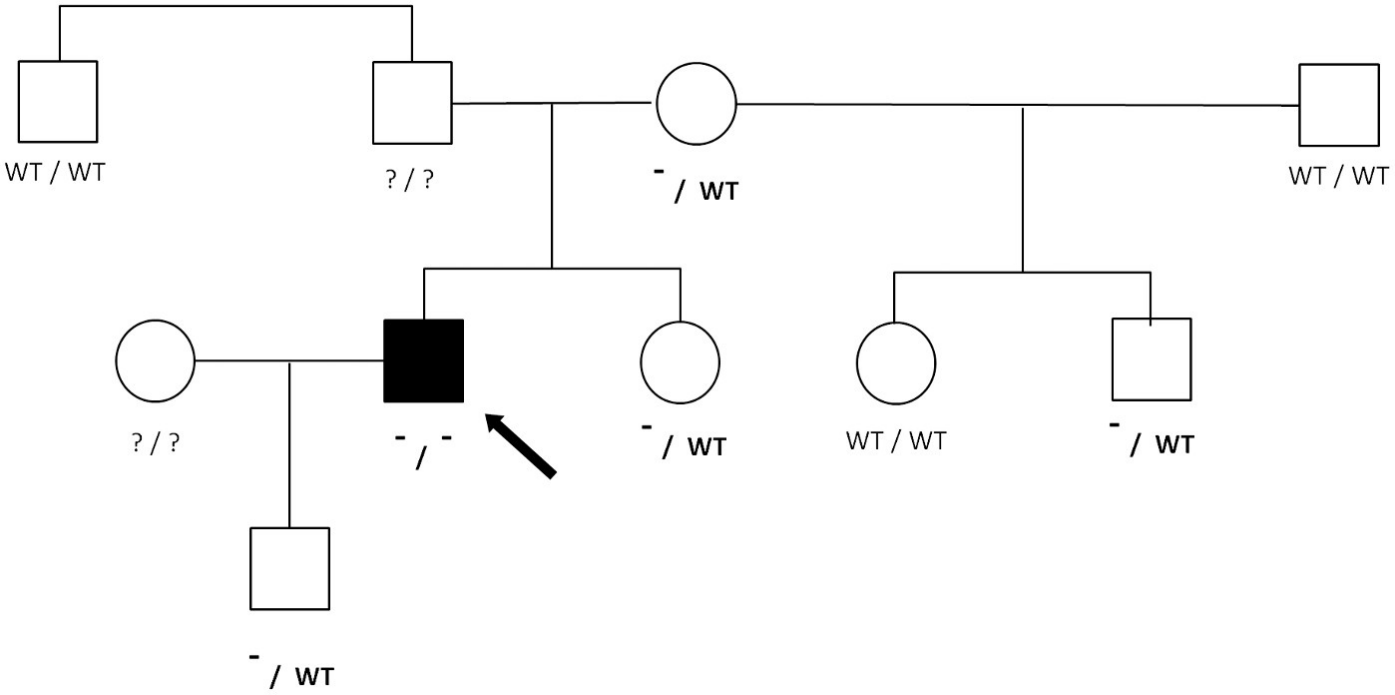
Pedigree chart of the proband BSS#1. Squares and circles denote males and females, respectively. The parents are connected by a horizontal line and a vertical line leads to their offspring. The filled symbol denotes an affected dog. The clear symbol denotes a healthy dog. The genotype of each dog is reported below the square or circle. WT: Wild-type allele; “-”: variant allele. The proband is indicated by an arrowhead.

The analysis of the platelet morphology was carried out using the ADVIA 2120 Hematology System (Siemens) in two (BSS#1 and BSS#3) out of the four probands and in the heterozygous relatives of BSS#1 (Fig 1).

This analyzer uses recent improvements in automated blood cell count and allows measurement of several platelet parameters, providing additional information regarding on the underlying mechanisms of thrombocytosis and thrombocytopenia. The platelets are scattered in a two-dimensional analysis based on the integration of a two-angle laser light scattering flow-cytometric method. By measuring the light scatter at a low angle scatter at 2–3° for volume assessment and a high angle scatter at 5–15° as refractive index, this instrument measures the following platelet parameters: platelet distribution width (PDW), platelet crit (PCT), mean platelet component (MPC), mean platelet mass (MPM), and large platelet count (LPLT; volume > 20fL), in addition to the total platelet count (PLT) and mean platelet volume (MPV). However, except for the PLT and MPV, these parameters are not routinely reported or widely used in clinical practice, and only limited information is available regarding dogs [12,13].

#### Western blot analysis

The dog platelets were lysed in sodium dodecyl sulfate (SDS) solution (Tris–HCl 50 mM pH 6.8; SDS 2%; glycerol 5%) supplemented with a cocktail of protease inhibitors (Sigma-Aldrich Co. LLC). The total protein contents were determined by the Lowry method using a protein assay kit (Sigma-Aldrich Co. LLC). Aliquots containing 10 μg of total proteins were separated using NuPage 4–12% bis-Tris Gel (Gibco-Life-Technologies) for 50 min at 200 V. The proteins were then electrophoretically transferred onto a nitrocellulose membrane using the Turbo Blot System (Bio-Rad). The blot was washed in PBS and protein transfer was checked by staining the nitro-cellulose membrane with 0.2% Ponceau Red and the gels with Comassie Blue. Non-specific binding on the nitrocellulose membranes was blocked with 5% milk powder in Phosphate Buffer Saline-0.1% Tween-20) (PBS-T20) for 1 h at room temperature. The membrane was then incubated over-night at 4°C with a 1:500 dilution of the primary monoclonal antibody mouse anti-CD42a (clone FMC-25, cod. MA5-16684, Thermo Fisher Scientific, Rockford, IL, USA). After several washings with PBS-T20, the membrane was incubated with the secondary biotin-conjugate antibody and then with a 1:1000 dilution of an anti-biotin horseradish peroxidase (HRP)-linked antibody.

The western blot was developed using a chemiluminescent substrate (Super Signal West Pico Chemiluminescent Substrate, Pierce Biotechnology, Inc, Rockford, IL, USA) according to the manufacturer’s instructions. The intensity of the luminescent signal of the resultant bands was acquired by Chemi Doc using Quantity One Software (Bio-Rad).

In order to normalize the CD42a data regarding the housekeeping protein, the membrane was stripped (briefly: the membrane was washed 5 min in water, 5 min in 0.2 M sodium hydroxide and then washed again in water) and re-probed for housekeeping β-tubulin (1:500 of anti β-tubulin sc-9104, Santa Cruz Biotechnology, USA). The relative protein content (cd42a/tubulin) was expressed as arbitrary units (AUs).

#### Immunocytochemistry

Blood samples in citrated tubes (Vacutest, KIMA, Italy) each containing 3.5 mL anticoagulated blood were collected from one of the authors (FG) as a positive control for the primary antibody, from one healthy dog (interspecies positive control) and from the BSS#1 affected dog. The blood tubes were centrifuged at 800 x g for 8 min at 4°C and the supernatant platelet rich plasma (PRP) was collected and transferred into 15 mL plastic tubes, and the platelets were counted. The PRP was then centrifuged again at 2000 x g for 5 min at 4°C. The supernatant was eliminated and the platelet pellet was gently resuspended in 1mL of PBS pH7.4, counted again and diluted using PBS pH7.4, it was counted at a final concentration of 1 x 10^3^ cells/μL. Two quantities of 10 and 100 uL of platelet resuspension were then immobilized on polylysinated slides by centrifuging for 10 minutes at 500 x g using a cytospin chamber in an Ettich centrifuge. Finally. the cytological smears were immediately frozen at -80°C and stored until use. The slides were hydrated in PBS and were processed for immunostaining. To block non-specific bindings, the sections were incubated in a solution containing 20% normal donkey serum (cod. 1700121, Jackson ImmunoResearch Laboratories, Inc, West Grove, PA, USA), 1% bovine serum albumin (cod. A-9418, Sigma Aldrich, Milan, Italy, Europe) in PBS for 1 h at RT. The slides were incubated overnight in a humid chamber at RT with the primary monoclonal antibody mouse anti-CD42a (clone FMC-25, cod. MA5-16684, Thermo Fisher Scientific, Rockford, IL, USA) diluted 1:200 in 1.8% sodium chloride (NaCl) in 0.01M PBS containing 0.1% sodium azide. After washing in PBS (3 x 10 min), the slices were incubated for 1 h at RT in a humid chamber with the secondary antibody goat anti-rabbit immunoglobulin G (IgG) fluorescein isothiocyanate conjugated (cod.401314; Merck Millipore, Darmstadt, Germany, Europe) diluted 1:200 in PBS. The same experiment was carried out permeabilizing the cell membranes by adding 1% Triton-X100 (cod. T8787, Sigma Aldrich, Milan, Italy, Europe). The slides were then washed in PBS (3 x 10 min) and mounted in buffered glycerol at pH 8.6 with 4',6-diamidino-2-phenylindole—DAPI- (Santa Cruz Biotechnology, CA, USA).

The slides were examined using a Nikon Eclipse Ni microscope equipped with the appropriate filter cubes to distinguish the fluorochromes employed. The images were recorded with a Nikon DS-Qi1Nc digital camera and NIS Elements software BR 4.20.01 (Nikon Instruments Europe BV, Amsterdam, Netherlands). Slight adjustments to contrast and brightness were made using Corel Photo Paint whereas the figure panels were prepared using Corel Draw (Corel Photo Paint and Corel Draw, Ottawa, ON, Canada).

#### Whole genome sequencing, variant calling

For the resequencing of one of the affected dogs, a PCR-free genomic fragment library with 350 bp insert size was prepared which, and collected roughly 28× coverage data on an Illumina HiSeq3000 instrument (2 × 150 bp). Read mapping, aligning and variant calling and filtering were carried out as previously described [14]. Functional candidate genes were visually inspected for structural variants using the BAM file from the affected dog and the Integrative Genomics Viewer [15].

#### Reference sequences

All the genetic analyses were carried out with respect to the CanFam 3.1 genome reference assembly and the NCBI Canis lupus familiaris annotation release 105. Numbering within the canine *GP9* gene refers to the mRNA accession number XM_846924.5 [16] and the protein accession number XP_852017.1 [17].

#### Genetic testing and epidemiological survey

**Fig 2:**
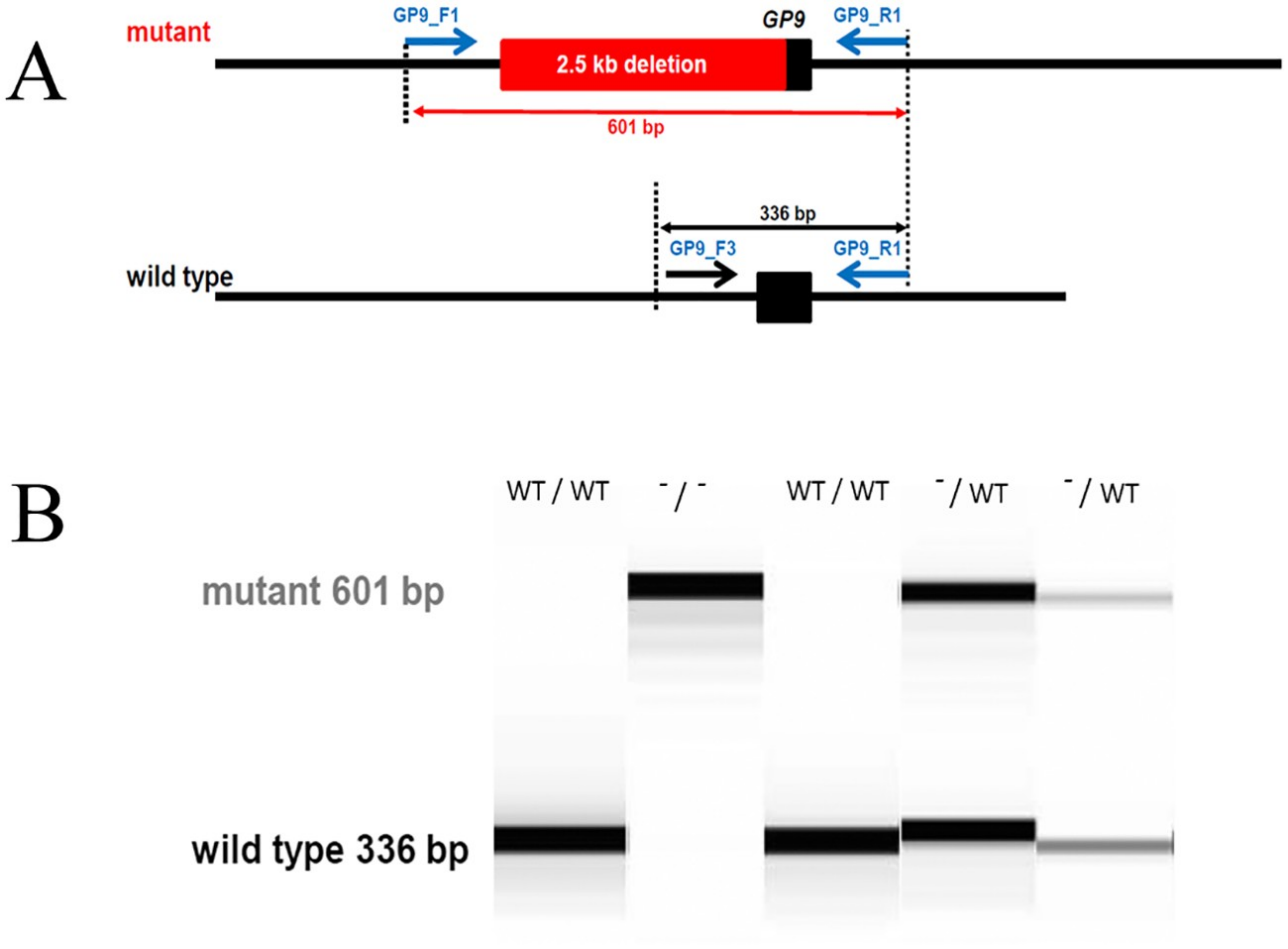
Genetic test assay. A) Schematic representation of the genetic testing assay; the common reverse primer GP9_R1 together with two different forward primers, GP9_F1 aligning in the conserved region upstream the *GP9* coding sequence and GP9_F3 aligning in the 2.5 kb deleted sequence are used to amplify in a multiplexed polymerase chain reaction assay the *GP9* locus. B) A representative examples of the results of the genotyping assay showing the 601 bp long and the 339 bp band long bands indicating the mutated and wild type alleles, respectively.

To ascertain the allele frequency in the CS population, an assay for the rapid detection of the deletion was established. To that aim, genetic testing was carried out using a multiplex end-point PCR. To that end, PCR was carried out using a mixture composed of 3 μL of 5× PCR buffer (Phusion GC Green buffer), 200 μM each dNTPs, 600 nM GP9_F1 primer (TAAGTGTGAAGCCGGTGAGC), 600 nM GP9_R1 (TACATCACGGCCACTGTCTC) primer, 400 nM GP9_F3 primer (CAGCCAGAGACGCAGGTAG), 0.3 u/reaction of Phusion Polymerase (Thermo Scientific), 2 μL of template, and molecular biology–grade water to reach a final volume of 15 μL. The PCR was carried out using a 3-step protocol: initial denaturation at 98°C for 30 s followed by 40 cycles at 98°C for 10 s, 63°C for 15 s, 72°C for an additional 15 s. The variant allele originates a band which is 601 bp long whereas the wild type allele originates a 339 bp band. (Fig 2).

Overall, ninety-eight gDNA samples of cocker spaniels were genotyped for the BSS variant. The gDNA was retrieved from two genetic testing laboratories (46 from Italy and 20 from Switzerland) and it was purified from additional thirty-two frozen blood samples harvested from CS examined for medical reasons at the Veterinary Teaching Hospital and which had been stored frozen in the laboratory hospital repository.

## Results and discussion

### Clinical manifestations

Clinical signs of diseases in the cases described herein overlapped those described in humans. Individual variation was remarkable. Bleeding tendencies were almost invariably present but were frequently overlooked. The most common sign indicating bleeding tendency was the hematoma formation after venipuncture reported by the referring practitioners; this occurred despite the careful and prolonged compression of the site after venipuncture which had been recommended in those dogs receiving the diagnosis. Episodic and self-limiting gingival bleeding were also frequently reported. Subtle signs of bleeding tendencies were overlooked until a trauma had exacerbated an overt hemorrhage. In the cases in the present study, trauma was minimal and had occurred during intense outdoor activity, nasal cavity endoscopic exploration following an episode of blood streaked nasal discharge or after mating. In addition, delivery was reported to be a likely inciting cause of uncontrolled bleeding. Bleeding was already evident in young dogs but not in puppies. The age at presentation for the first evidence of a bleeding problem was 2–4 years. In humans, bleeding tendencies associated with BSS are usually evident from early childhood. However, the severity of symptoms may progressively worsen or ease throughout puberty and adult life according to different variants [6]. The cases in the present study had been monitored for a few years. No conclusion could be drawn to support a worsening of bleeding signs over time. Instead, the signs seemed to be stable and the dogs continued to experience a good quality of life. Also, in the case series reported here, the majority of dogs were male. However, no gender predisposition is reported in human medicine and only the increased mortality risk associated with surgery in women is reported [18].

The owners have to be warned that their dogs will suffer bleeding tendencies throughout their entire life and that the only recommended therapy in case of severe hemorrhage is whole blood or platelet rich plasma from a fully compatible dog. Since it is likely that transfusion will have to be repeated during the lifetime of the dogs, the blood group typing of both the donor and the affected dog was imperative. The use of DDAVP (1-deamino-8-D-arginine vasopressin) has been reported to improve the clinical signs and to shorten the time of bleeding in some human patients [19–22]. The underlying mechanism is still unknown but it might be related to an increase in factor FVIII levels induced by DDAVP infusion [23]. Another recommendation is to cautiously use drugs having an inhibitory effect on platelet aggregation, such as aspirin or, more generally, non-steroid anti-inflammatory drugs. Clearly, immunosuppressive therapy for IMT is detrimental and should be avoided.

In humans, BSS is characterized by a bleeding tendency, circulating giant platelets in the blood and variably low platelet counts. Clinical manifestations usually include purpura, epistaxis, menorrhagia, and gingival and gastrointestinal bleeding. In humans, the diagnosis is based on a prolonged skin bleeding time, the presence of a small number of very large platelets (macrothrombocytopenia), defective ristocetin-induced platelet agglutination and/or low or absent expression of the GPIb-V-IX complex as evidenced by flow cytometry. Prothrombin consumption is markedly reduced. However, the disease is listed among a group of disorders of platelets with overlapping clinical pictures. For these orphan (rare) diseases the diagnosis is very challenging and frequently delayed or even completely missed in particular in developing countries [24,25]. To overcome this drawback, third level referral centers may use refined diagnostic protocols which include combination of basic methods, such as platelet morphology and immunofluorescence carried out directly on a blood smear with available genetic testing to diagnose hereditary platelet disorders. The prognosis is usually good with adequate supportive care but severe bleeding episodes can occur with trauma and surgical procedures especially in those subjects in which a diagnosis has not yet been established. Treatment of bleeding or prophylaxis during surgical procedures usually requires platelet transfusion, desmopressin and antifibrinolytics [18].

### CBC phenotype

The platelets of the proband dogs BSS#1 and BSS#3 were characterized using platelet indices measured by the ADVIA 2120. Both dogs presented macrothrombocytopenia with variable platelet counts over time even in the same subjects (Table 1).

**Table 1:** Platelet indices measured by ADVIA 2120.

	BSS#1 dog		Heterozygous relatives; n = 4		BSS#3 dog	Reference population; n = 1928		ADVIA 2120 Canine Reference interval ^§^
	median	range	median	range		median	5^th^–95^th^ percentiles	
**PLT** **(10**^**3**^ **cells/μL)**	**56.00**	42.00–125.00	**321.50**	240.00–340.00	**49.00**	**325.00**	108.05–689.95	173.0–486.0
**PCT(%)**	**0.16**	0.13–0.37	**0.36**	0.30–0.42	**0.11**	**0,40**	0.05–1.23	0.3–0.5
**MPV(fL)**	**27.50**	24.10–30.90	**12.15**	9.70–13.90	**24.20**	**12,5**	9.0–19.2	8.56–14.41
**Large PLT** **(10**^**3**^ **cells/μL)**	**33.00**	28.00–81.00	**23.50**	12,00–33,00	**26.00**	**35.5**	7.0–113.7	6.64–84.23
**PDW(%)**	**52.95**	49.40–61.70	**62.35**	57.70–65.20	**58.90**	**64,7**	52.84–73.07	55.7–66.9
**MPC(g/dL)**	**21.25**	20.60–24.00	**22.20**	22.00–25.00	**21.80**	**20,9**	17.4–24.2	14.0–18.6
**PCDW(g/dL)**	**6.85**	4.40–7.90	**4.60**	4,30–5,70	**8.00**	**6.8**	3.9–8.3	4.58–7.05
**MPM(pg)**	**4.02**	3.64–4.13	**2.45**	2,26–2,64	**3.05**	**2.2**	1.8–3.0	1.32–1.92
**PMDW(pg)**	**1.11**	0.91–1.61	**0.98**	0,88–1,07	**1.22**	**0.9**	0.7–1.3	0.51–0.84

PLT: platelet count; PCT: thrombocrit; MPV: mean platelet volume; Large platelet: count of platelets with a volume > 20fL; PDW: platelet distribution width; MPC: mean platelet component concentration; PCDW: mean platelet component distribution width: MPM: mean platelet mass; PMDW: mean platelet mass distribution width.

^§^ Moritz et al., 2004

**Fig 3:**
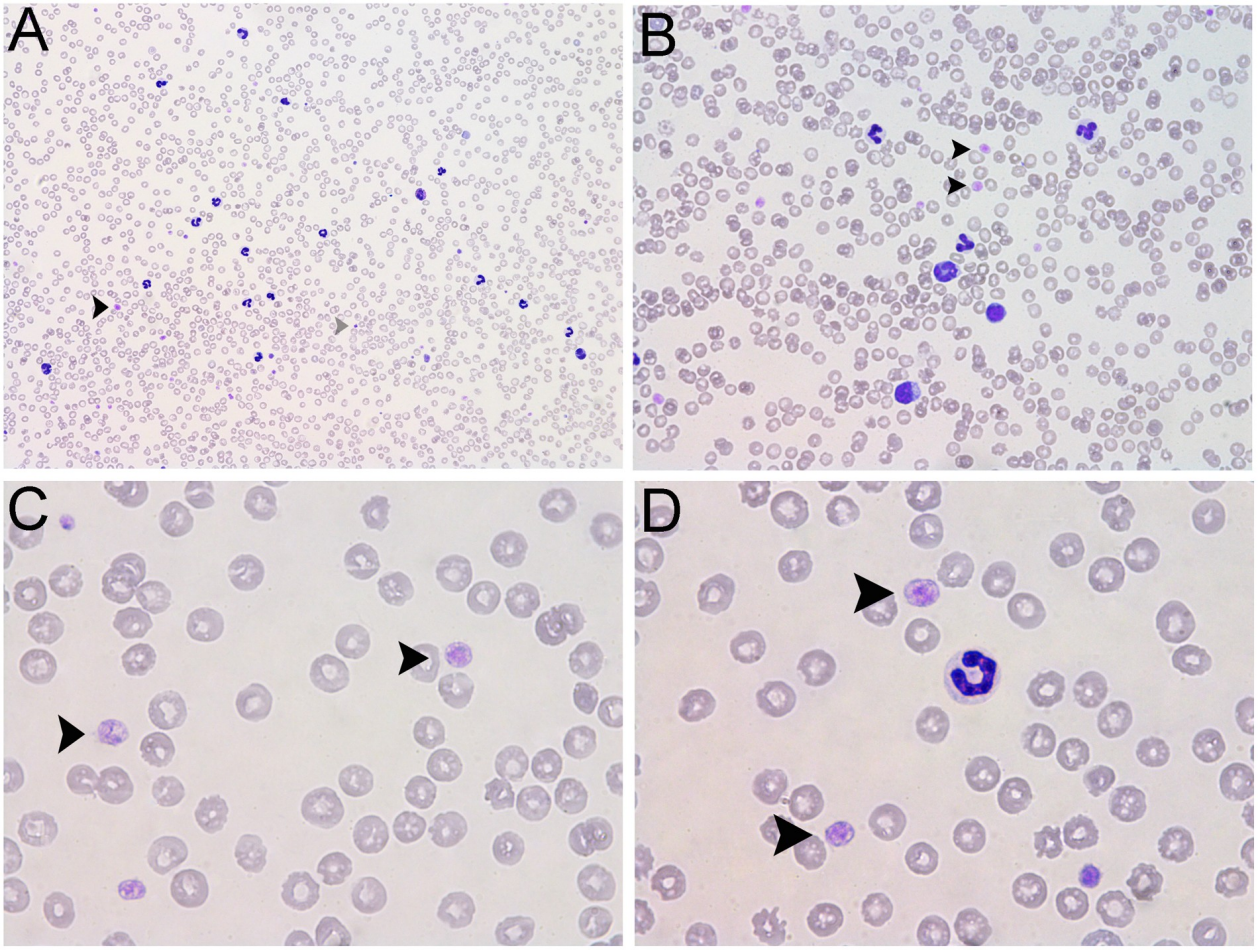
Platelets morphology in a Bernard-Soulier syndrome affected dog. Light microscopy of May Grünwald-Giemsa stained smears of the BSS#1 affected dog at 100 X (A); 400 X (B) and 1000 X (C,D) magnification. Black arrowheads indicate “giant platelets” in comparison to normal platelets (grey arrowhead).

Almost half of the platelets were markedly large as observed on May Grünwald-Giemsa (MGG) stained smears, even approximating the red blood cell (RBC) volume (Fig 3).

In humans, a mean platelet diameter > 3.7 μm is considered the cutoff of “giant platelets” typical of Bernard-Soulier syndrome and MYH9 disorders. Similarly, the size of “giant platelets” in dogs was between half and one RBC in diameter (6–8 μm) [26]. The presence of “giant” platelets was confirmed by an elevated count of “large platelets” having a volume > 20fL. From 47 to 67% of the total count were large platelets (Table 1). Although half of the platelets were larger than normal, the platelet distribution width (PDW) index, which mirrors the variability of platelet size, was lower than normal (Table 1). In fact, the PDW histograms of the affected dogs indicated that the platelets aggregated in 2 clusters with low and high volume, respectively with a pattern different from the typical anisocytosis seen with a high PDW.

In humans, it has been reported that platelets concentrations fluctuate widely [6] over time between marginally low (30000/μL) to normal values even in the same subject. The probands herein described presented PLT values which were always below the threshold of the reference range. However, the plateletcrit may occasionally fall within the reference range (Table 1) since the whole platelet mass takes into account the low number but also the higher than normal volume of platelets.

The giant platelet granular density appeared quite normal and no cytoplasmic projection of pseudopods indicating activation could be seen (Fig 3). This was additionally confirmed by the higher than normal mean platelet component (MPC) which is a measurement of the mean refractive index. The MPC indicates platelet density, and decreases after activation and subsequent degranulation. In dogs, the MPC has been shown to decrease during long storage activation [13].

In veterinary medicine, very little information is present in the literature regarding the platelet indices in dogs. Platelet indices have been evaluated in endotoxiemia [27], IMT [28], babesiosis [29], and sample storage [13], and in healthy dogs for reference range establishment [12].

Based on the platelet indices measured in the probands, a step-by-step algorithm was implemented (S2 Fig) and used to search for other possible hereditary macrothrombocytopenias in the CBC archive of the Veterinary Teaching Hospital of the University of Bologna over a one-year period. Of the 1940 CBCs analyzed over the course of one year and excluding the CBCs of the BSS#1 affected dog, only eight cases were selected by the algorithm, five being Cavalier King Charles Spaniel dogs, a hemolytic mongrel dog affected by pyometra, a case of Ehrlichiosis and a case of immune-mediated hemolytic anemia and IMT. The data from a more numerous group of affected dogs could certainly have refined the thresholds and the overall usefulness of an algorithm based on platelet indices. Furthermore, the algorithm may be helpful in differentiatinmg hereditary macrothrombocytopenias from acquired ones.

### Molecular basis

Based upon the evaluation of PLT indices and the morphology of those subjects included in the pedigree of proband BSS#1, the heterozygous relatives (Fig 2) did not show any differences in any parameter with respect to the reference population [30]. Hence, it is very likely that the disease shows an autosomic recessive inheritance pattern. In humans, almost all the cases reported alsos1 show an autosomal recessive inheritance, and only a few families having autosomal dominant forms with variants in the *GP1Bα* gene [31,32] or *GP1Bβ* have been described [33].

**Fig 4:**
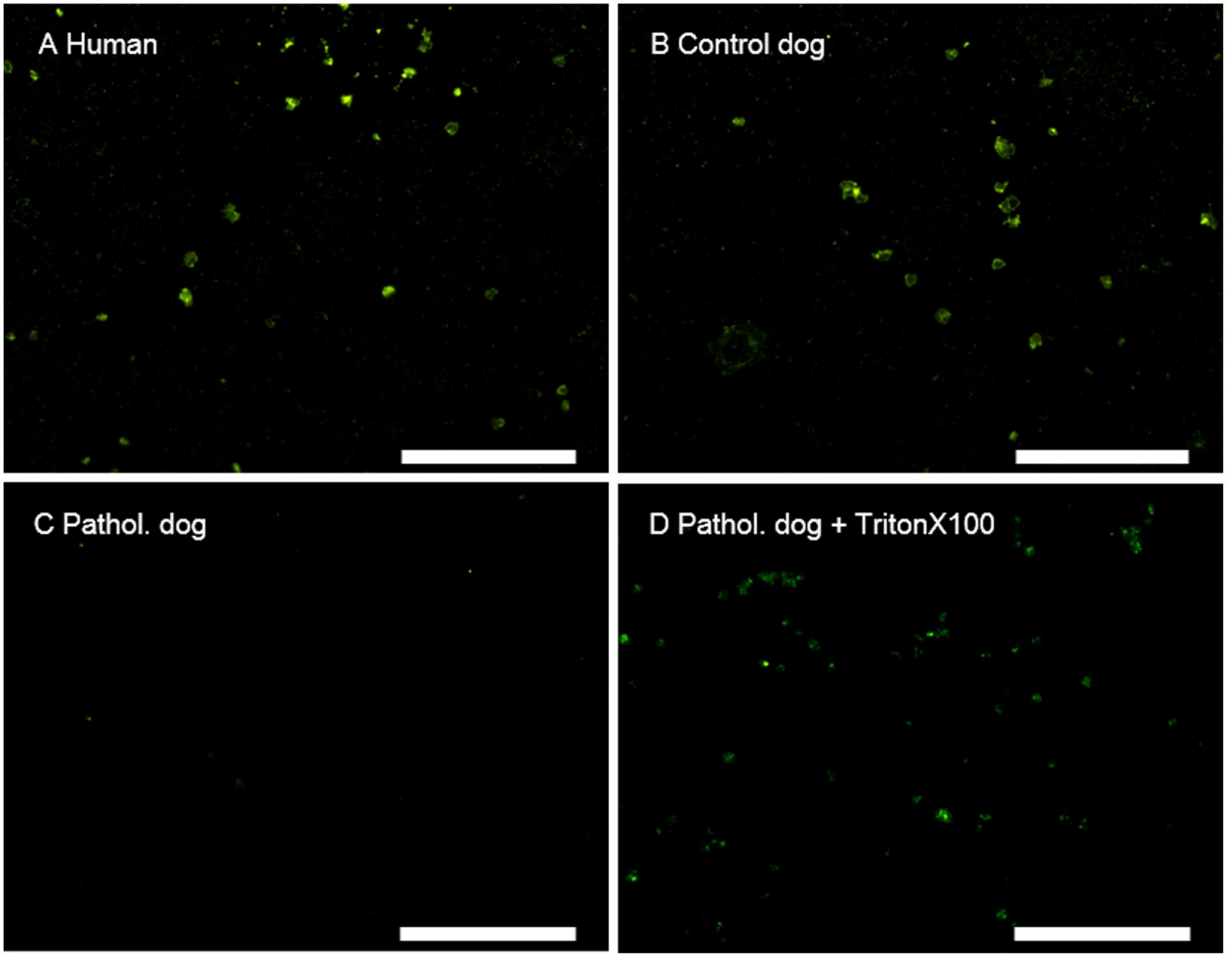
Platelet immunocytochemistry. Photomicrographs showing CD42a immunoreactivity. In human (A) and in healthy dog (control) platelet samples (B), a strong signal was evident on the cell membranes. In the sample from the BSS#1 affected dog (C), no signal was detected. Under permeabilization conditions (Triton X100), the pathological sample shows an intracellular signal (filled arrows) (D). Scale bars = 50 um.

The proband BSS#1 showed the lack of the GPIb-V-IX complex on the platelet cell membrane. In platelets from the healthy human volunteer and the healthy dog (positive controls), CD42a was strongly expressed on the cell membrane while, in the BSS#1, no signal was detected, demonstrating the lack of CD42a assembled in the GPIb-V-IX complex at the platelet membrane (Fig 4).

The lack of functional GPIb-IX-V on the platelet membrane is the hallmark of all the BSS variants reported in humans, and results in impaired binding to vWF and a dramatically reduced ability to adhere to the subendothelium of a damaged vascular wall or to agglutinate in response to ristocetin. Furthermore, there is the absence of other binding sites for P-selectin, thrombospondin-1, factor VIIa, factor XI, factor XII, aMb2, and high molecular weight kininogen. While the majority of the functional domains and the binding sites for vWF and thrombin are on GP1bα, both GP1bβ and GPIX are necessary for the adequate trafficking of the GPIb-V-IX complex and its expression on the cell membrane by hampering its degradation [34,35].

Whole genome sequencing of one affected dog and subsequent automated variant filtering based on a monogenic autosomal recessive mode of inheritance with respect to 188 non-affected control dogs of different breeds and three wolves, revealed 1927 homozygous variants private to the affected dog. None of these variants was located within a candidate gene. Given that the automated pipeline did not detect any private protein changing variants affecting a gene known for a platelet disorder, it was hypothesized that a larger structural variant might be causative for the disorder. Structural variants, such as large insertions, deletions, duplications, or inversions would be missed by the applied variant detection software. Therefore, all four known genes (*GP1BA*, *GP1BB*, *GP5*, and *GP9*) involved in human BSS were selected as functional candidate genes, and they were visually inspected for structural variants.

**Fig 5:**
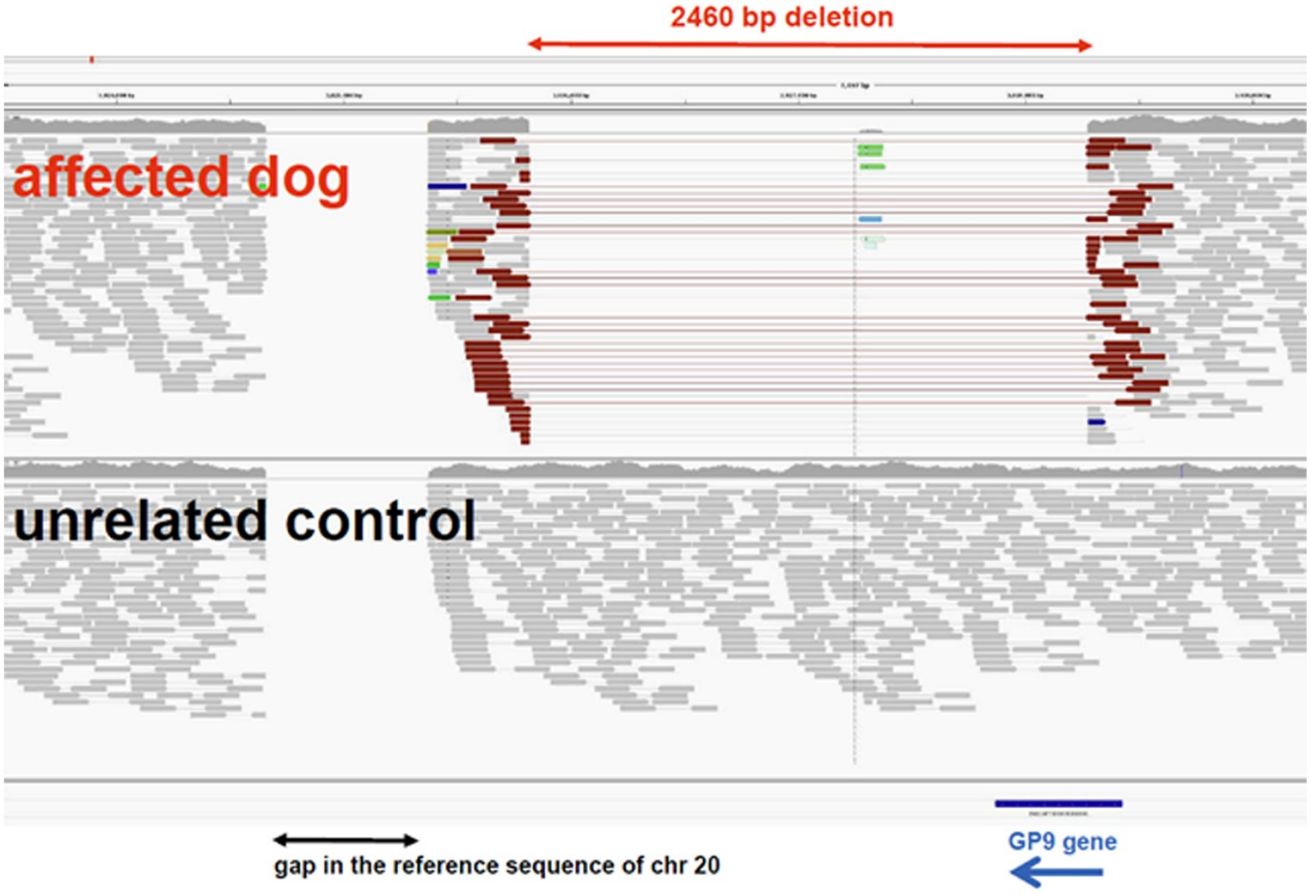
Large genomic deletion spanning the coding exon of the canine *GP9* gene in the BSS-affected dog.

In the chromosomal region of the *GP9* gene, a large structural variant was detected in homozygous state in the genome of the affected dog (Fig 5).

The formal variant designation is chr20:3,025,814_3,028,273del2460. The deletion truncates 104 (71%) of the 146 codons of the wildtype canine reading frame. No structural variants affecting the coding regions of the other candidate genes were detected.

The lack of the GPIb-V-IX complex in the BSS cases herein described in Cocker Spaniel dogs was most likely caused by a large 2460 bp deletion, spanning almost half of *GP9* exon 2. Exon 2 is the only coding exon of the *GP9* gene, which also encompasses a small leading exon 1. Variants on *GP9*, such as homozygous [36] or compound heterozygous with other *GP9* or *GP1BB* and *GP1BA* gene have been reported in humans.

To confirm the presence of the deletion, a PCR approach was chosen. Two primers flanking the large deletion were designed as well as one primer inside the deletion, and a PCR was carried out using these three primers. In the affected dogs, a single PCR product resulted and, for the heterozygous relatives two PCR products resulted, which was consistent with the expected fragment sizes (Fig 2).

Despite the deletion of the C-terminus, the GPIX was detected with WB in platelet lysates at an approximate 50% level of expression in comparison to dogs carrying wild-type alleles in homozygousity (S3 Fig); even so, the deleted protein was expressed but was not localized with the other proteins on the cell surface as GPIb-V-IX complex. In fact, using the same monoclonal antibody as in WB and permeabilization with Triton X-100 added to the solutions, immunostaining of naïve platelets of the affected dog revealed a weak signal of CD42a with a patchy pattern consisting of intracellular aggregates and a faint signal from the membrane. In human BSS variants, variants occurring in *GPIX* prevent the expression of the complex at the cell surface, markedly reducing the overall protein expression, which may accumulate in the endoplasmic reticulum and (ER) and ER-associated Golgi [34,37]. The vast majority of GPIX variants which have been reported until now are missense [8] while GPIX protein truncation determined by frameshift (insertions, duplication/ small deletions) or nonsense variants have been reported very rarely [38–41]. In one case, N-terminal truncated GPIX protein expression was almost completely absent on cell membrane and also intracellularly [40]; in other cases, the affected platelets were only investigated using flow cytometry, and assumptions on the intracellular localization and accumulation could not be drawn. In the present study, the deletion may have bridged the remaining 5’ part of coding exon 2 with sequences downstream exon. *In silico evaluation* showed that the novel stop codon may occur after that 58 novel aa are included in translation. The variant protein would be 144 aa long instead of 177. The theoretical molecular weight (MW) of the two aa chains differ of three Kda. This small MW difference may explain why WB found an apparently specific band also in the affected dog.

WB analysis showed a band corresponding to CD42a in both the wild type and variant samples, however the intensity was significantly reduced in pathological sample which in turn appeared slightly shifted downward.

### Allele prevalence

Based on the findings of the genetic survey the prevalence of the variant allele was 4.6% with a distribution of genotypes frequencies of 91.8% of homozygous wild-type, 7.1% of heterozygous and 1% of homozygous variant. The genotype frequencies were consistent with the Hardy-Weinberg equilibrium (chi-squared = 3.35; p = 0.07).

## Conclusions

In conclusion, a novel hereditary bleeding disorder affecting Cocker spaniel dogs and the responsible variant thereof has herein been described for the first time. The disease is the orthologue of BSS described in humans and could represent a valid animal model of spontaneous disease. To date, BSS has never been reported in dogs nor in any other domestic animals. Recently, the International Mouse Phenotyping Consortium reported that the knockout of *Gp9* in mice caused thrombocytopenia with increased platelet volume [42].

Furthermore, the genetic test could support the diagnosis in those dogs with bleeding tendencies or ongoing thrombocytopenia in addition to an ancillary test of clinical pathology, such as light microscopy, CBC including PLT indices, immunofluorescence and/or cytometry. For those dogs, having a diagnosis of BSS, appropriate support in terms of genetic counselling would be warranted in order to guarantee an adequate quality of life and life expectancy. Finally, a genetic test could easily identify carrier dogs and be introduced as a marker assisted breeding programs/genetic counselling.

## Supporting information

S1 Fig Bleeding in BSS#1 affected Cocker Spaniel dog. (TIF)

S2 Fig Algorithm aimed at diagnosing Bernard-Soulier syndrome affected dogs using ADVIA 2120, Siemens. (TIF)

S3 Fig Western blot analysis in a healthy control dog and a Bernard-Soulier syndrome affected dog. (TIF)
