# Supplementary figures and images for: A large deletion in the GP9 gene in Cocker Spaniel dogs with Bernard-Soulier syndrome
Source: PLoS One. 2019 Sep 4;14(9):e0220625. doi: 10.1371/journal.pone.0220625 (PMC6726462; doi:10.1371/journal.pone.0220625)

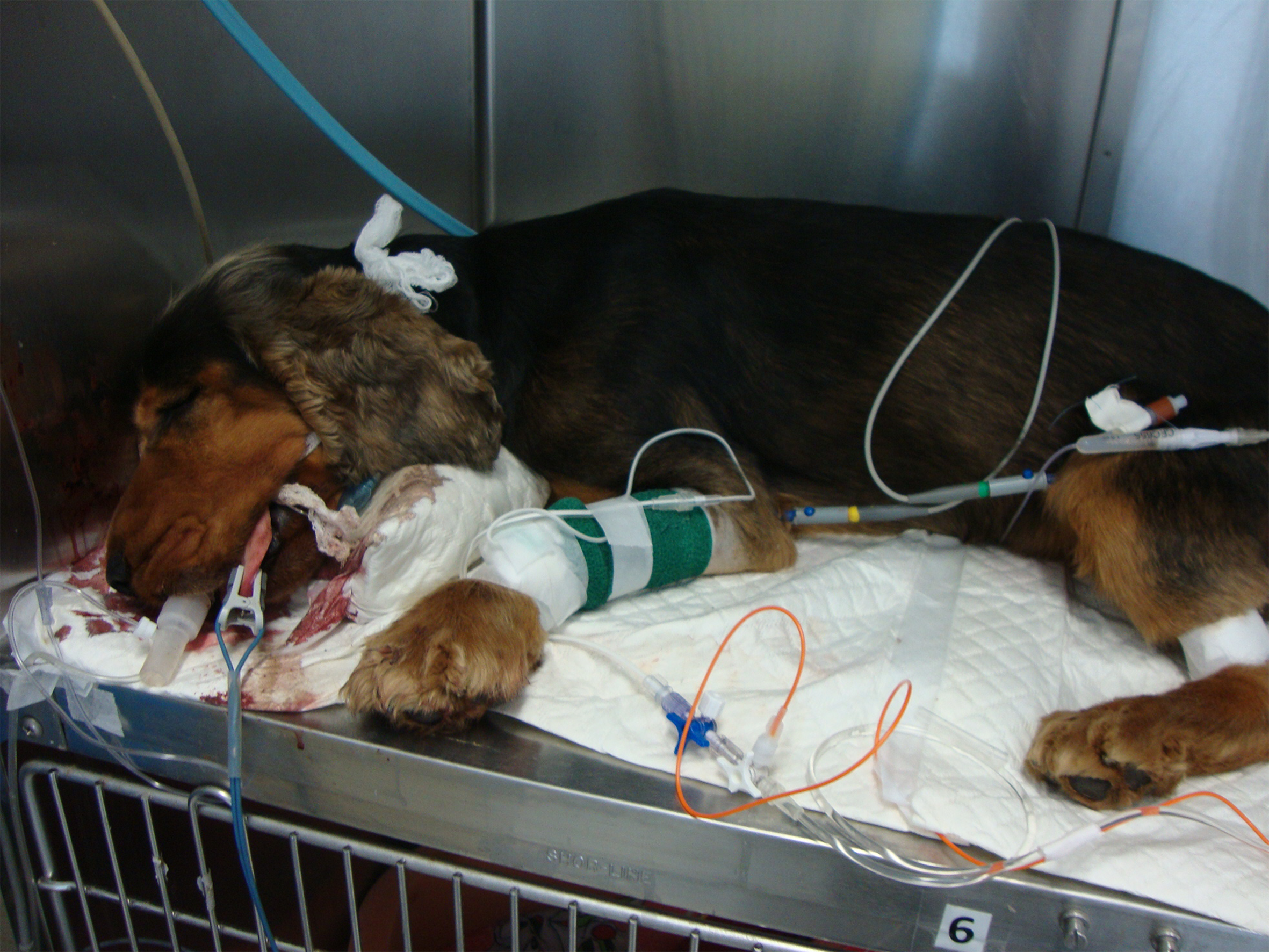

Supplement: S1 Fig — (TIF) [file pone.0220625.s001.tif]

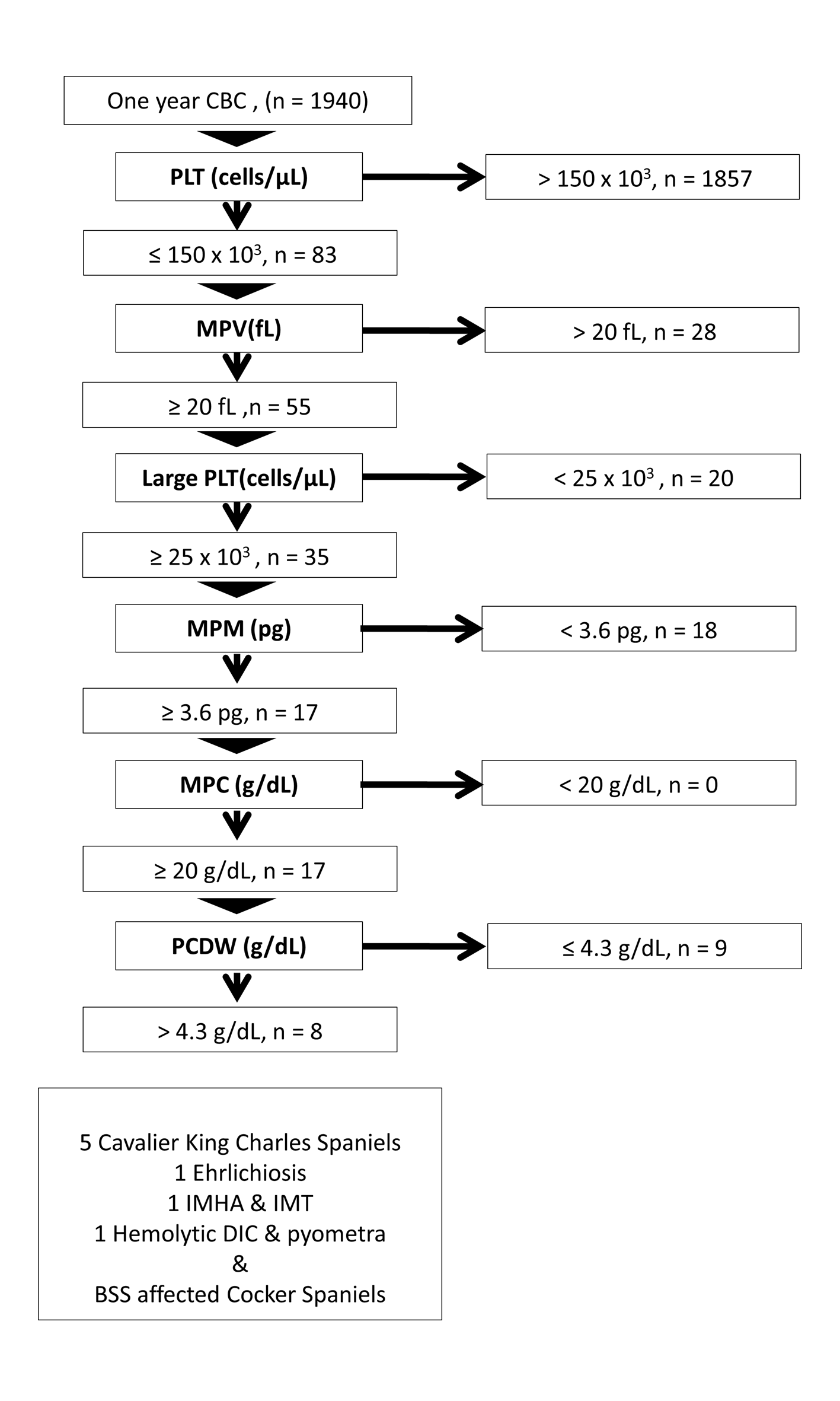

Supplement: S2 Fig — (TIF) [file pone.0220625.s002.tif]

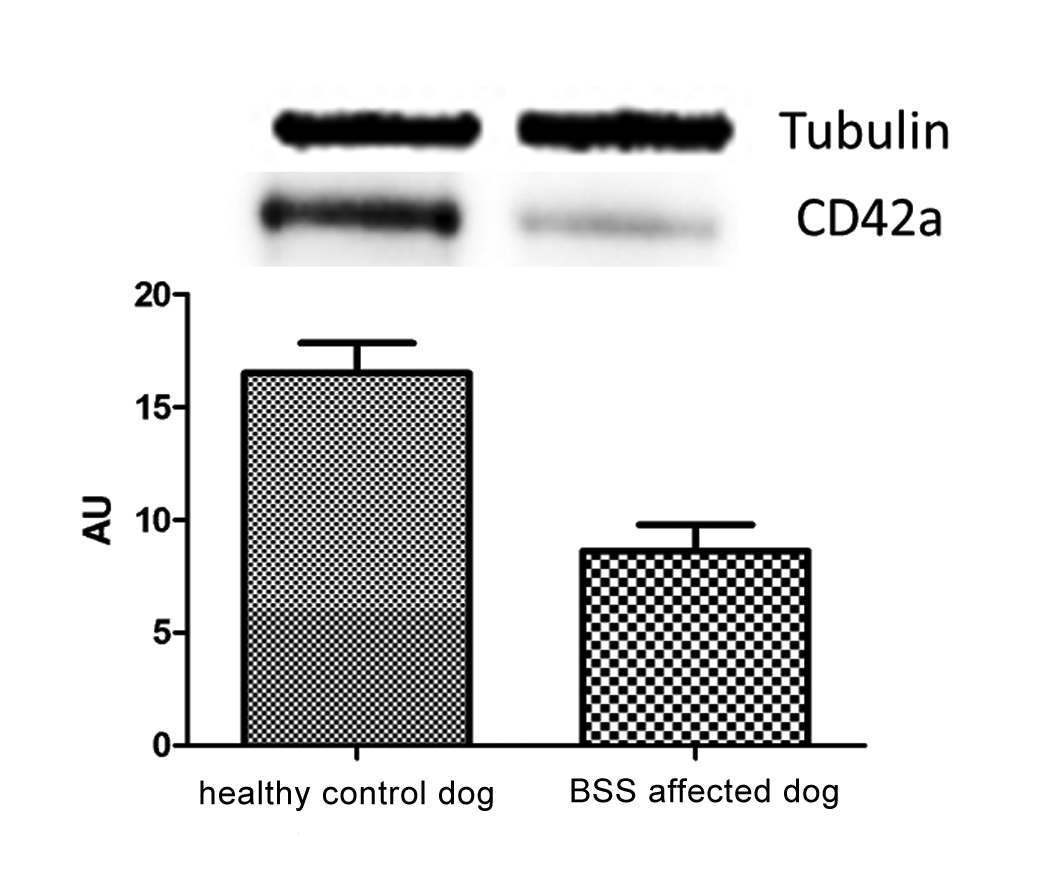

Supplement: S3 Fig — (TIF) [file pone.0220625.s003.tif]
